# Spatial and Temporal Variability of Ambient Underwater Sound in the Baltic Sea

**DOI:** 10.1038/s41598-019-48891-x

**Published:** 2019-09-13

**Authors:** Mirko Mustonen, Aleksander Klauson, Mathias Andersson, Dominique Clorennec, Thomas Folegot, Radomił Koza, Jukka Pajala, Leif Persson, Jarosław Tegowski, Jakob Tougaard, Magnus Wahlberg, Peter Sigray

**Affiliations:** 1 Tallinn University of Technology, School of Engineering, Tallinn, 19086 Estonia; 2 Swedish Defence Research Agency, Stockholm, SE-164 90 Sweden; 3 Quiet-Oceans, Plouzané, 29280 France; 4 Finnish Environment Institute, Helsinki, FI-00251 Finland; 5 University of Gdansk, Institute of Oceanography, Gdynia, 81-378 Poland; 6 Aarhus University, Department of Bioscience, Roskilde, 4000 Denmark; 7 University of Southern Denmark, Department of Biology, Odense M, 5230 Denmark

**Keywords:** Ecology, Environmental impact, Physical sciences

## Abstract

During last decades, anthropogenic underwater sound and its chronic impact on marine species have been recognised as an environmental protection challenge. At the same time, studies on the spatial and temporal variability of ambient sound, and how it is affected by biotic, abiotic and anthropogenic factors are lacking. This paper presents analysis of a large-scale and long-term underwater sound monitoring in the Baltic Sea. Throughout the year 2014, sound was monitored in 36 Baltic Sea locations. Selected locations covered different natural conditions and ship traffic intensities. The 63 Hz, 125 Hz and 2 kHz one-third octave band sound pressure levels were calculated and analysed. The levels varied significantly from one monitoring location to another. The annual median sound pressure level of the quietest and the loudest location differed almost 50 dB in the 63 Hz one-third octave band. Largest difference in the monthly medians was 15 dB in 63 Hz one-third octave band. The same monitoring locations annual estimated probability density functions for two yearly periods show strong similarity. The data variability grows as the averaging time period is reduced. Maritime traffic elevates the ambient sound levels in many areas of the Baltic Sea during extensive time periods.

## Introduction

The influence of the sounds from increasing human activities pose considerable risks for the overall health of our seas. Anthropogenic underwater sound is recognized as a pollutant that may have long-term detrimental effects on marine ecosystems. Among these effects are the reduction of communication space^1–3^, increase in stress levels and perturbation of development of marine species^4^. At the same time, the list of marine animals known to be sensitive to sound has been ever extending. Now it is ranging from marine mammals and fishes to crustaceans and invertebrates. As an example, cod can perceive noise generated within a frequency range of 100–1000 Hz and display a heightened cortisol plasma level with the potential negative impacts on their spawning performance^5^. Another recent study demonstrated reduced foraging in harbour porpoises caused by loud ship noise^6^.

EU’s Marine Strategy Framework Directive (MSFD) was adopted in June 2008 with the aim to achieve Good Environmental Status (GES) and maintaining the marine biodiversity of European marine habitats by the year 2020^7^. The directive sets qualitative descriptors for GES. Among these, Descriptor 11 (D11) concerns energy introduced into the marine environment, including underwater sound, which should be at levels that do not adversely affect the marine environment. The Technical Sub-Group on Underwater Noise (TSG Noise) issued a set of recommendations^8^ and monitoring guidance specifications^9^. Another monitoring guide has been issued by NPL^10^. Concerning the criterion for the continuous low-frequency underwater sound, it is stated that it should be monitored in two one-third octave bands (here and after base 2) with the center frequencies of 63 Hz and 125 Hz^11^. The sound pressure level (SPL) of these frequency bands has been chosen due to being a proxy for the abundance of the continuous low-frequency anthropogenic sound, mostly generated by commercial vessels.

Setting up sound monitoring programmes is the first step in the assessment of the levels of anthropogenic underwater sound in marine habitats. Initially, they help to establish the baseline levels of sound. Both deep-ocean observatories^12,13^ and autonomous recording systems^14–16^ have been used for monitoring. Ideally the monitoring of underwater sound can be imagined to be a network of cabled monitoring stations that sufficiently cover a given marine area. However, costs limit this kind of ambition and a realistic monitoring programme will entail only a few monitoring locations. This has the drawback of not being representative of the whole marine area. In order to circumvent this limitation, sound propagation modelling is commonly used in combination with the monitoring. Modelling helps to estimate the spatial extent of sounds from various anthropogenic sources (mainly ships) in a given marine environment^17,18^.

In order to assess the levels of underwater sound in a big marine area that is the Baltic Sea a joint international cross-bordering effort is needed. First project of this kind was Life+ “Baltic Sea Information on the Acoustic Soundscape“ (BIAS) launched in 2012^19^. The main aim of the BIAS project was the characterisation of the Baltic Sea soundscape. The modeled soundscape was mapped and a planning tool for using the maps developed^20^. Therefore, the monitored sound data was primarily used to calibrate and ground truth the sound propagation modelling. Although the modelling provides information about spatial sound level distribution, the data gathered during a sound monitoring programme are potentially a valuable source of additional information. When compared to modelling, it possesses higher resolution and is the most accurate representation of the actual sound levels in a location. Therefore, the data from sound monitoring in a marine area are worthy of being analysed separately and more thoroughly. There is a large number of open questions related to long-term monitoring of sound that need to be answered. Consequently, this paper aims to provide a detailed analysis of the extensive BIAS sound monitoring data. Moreover, the recorded data can serve as a baseline of the Baltic Sea underwater ambient sound that can be used for tracking changes in the following years.

## BIAS-project and Sound Monitoring Locations

**Figure 1:**
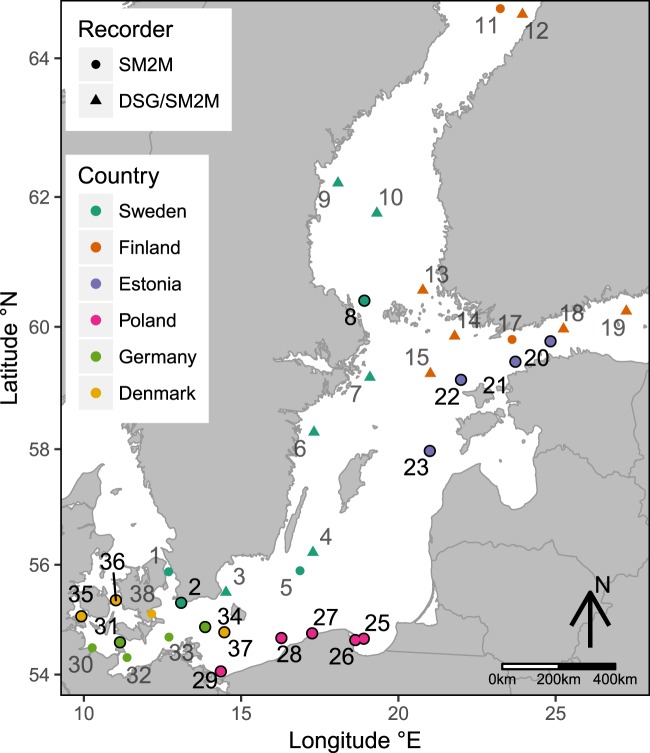
Underwater sound monitoring locations of the BIAS-project. The locations where SM2M marine submersible recorder was used throughout the sound monitoring are depicted with a circular marker and locations with alternate deployments of the DSG Ocean recorder with triangular markers. More details on the measurement equipment can be found in the methods section of this paper. The numbers of the monitoring locations analysed in this paper are highlighted with a darker colour.

An extensive BIAS sound monitoring programme was launched by the joint effort of scientists from six Baltic Sea countries - Sweden, Denmark, Germany, Poland, Estonia, and Finland. In shallow watered seas this programme stands out for its longevity and spatial coverage. Figure 1 shows the sound monitoring locations of the BIAS-project. The locations were chosen to cover different depths, seabed substrates and ship traffic intensities. The numbering of the monitoring locations follows a clockwise direction around the coast of the Baltic Sea. The numbering starts in the Øresund Strait near the coast of southern Sweden and finishes with location number 38 in the Faxe Bay, Denmark. A large amount of data has been collected from all these measurement locations during the period from 1 January 2014 to 31 December 2014. For the sake of brevity we will restrict the discussion and analysis to the data from some specific but spatially representative monitoring locations listed in Table 1. This table shows that the water depth of the locations varies from 12 m to 89 m and a variety of seabed substrates. Ice cover did not occur in the selected monitoring locations during the winter of 2014. This was affirmed by the SMHI open-access data from HIROMB BS01 oceanographic forecast model.

**Table 1:** Selected monitoring locations from the BIAS-project.

No.	Country, Loc. Name	Lat. °N	Lon. °E	Depth [m], Seabed Substrate
2	SWE-Trelleborg	55.3210	13.0950	23, Coarse substrate
8*	SWE-Sea of Åland	60.4158	18.9183	45, Mixed Sediment
20	EST-GoF, Tallinn	59.7715	24.8397	75, Mud to Muddy Sand
21	EST-GoF, Paldiski	59.4416	23.7276	89, Mud to Muddy Sand
22	EST-Hiiumaa	59.1499	21.9901	70, Mud to Muddy Sand
23	EST-Saaremaa	57.9689	21.0035	88, Mud to Muddy Sand
25	POL-Gulf of Gdansk	54.6665	18.9001	80, Mud to Muddy Sand
26	POL-Puck Bay	54.6413	18.6310	30, Mud to Muddy Sand
27	POL-Łeba & Rowy	54.7649	17.2589	18, Sand
28	POL-Darłowo-Ustka	54.6793	16.2813	41, Coarse substrate
29	POL-Świnoujście	54.0602	14.3549	12, Sand
31	GER-Fehmarn Belt	54.5997	11.1497	27, Sand/Mud to M-Sand
34	GER-Arkona Basin	54.8803	13.8574	44, Mud to Muddy Sand
35	DNK-Little Belt	55.0755	9.92133	30, Mud to Muddy Sand
36	DNK-Great Belt	55.3672	11.0193	27, Mixed/Mud to M-Sand
37	DNK-Rønne Banke	54.7853	14.4673	20, Sand/Coarse substrate

Seabed substrate data originates from EMODnet (http://www.emodnet-geology.eu/). The asterisk behind location 8 is to point out significantly lower data coverage (May to December 2014, coverage 64% against 88–100% for the other locations).

**Figure 2:**
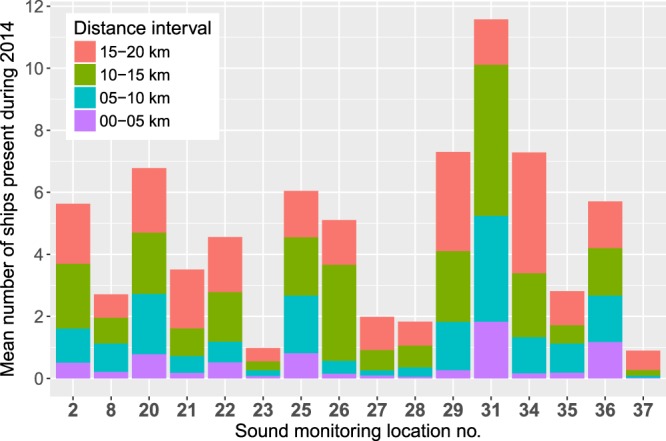
Average ship traffic intensity at different distance intervals within 20 km maximum range from the monitoring locations in 2014. The intensity calculation is based on 20 second time-regularised ship location data from the automatic identification system (AIS). The mean number of ships corresponds to the most likely number of ships within a distance interval at a randomly selected time during 2014.

The distance to the closest shipping lane as well as the intensity of ship traffic are two important parameters that affect the continuous low frequency anthropogenic sound levels. The proxy of the shipping intensity was taken to be the normalised number of time regularised automatic identification system (AIS) ship distances from the monitoring location. The AIS data for the BIAS project was provided by the Helsinki Commission (HELCOM). The time regularisation of the AIS data was necessary as the intervals for the systems location reporting are irregular in time. The reporting frequency is dependant on the rate of turn and speed of the ships as well as the class of AIS transceiver. The time regularised AIS ship distances were normalised to give the mean number of ships most likely to be present at any time during 2014. Figure 2 shows the differences in the mean number of ships near monitoring locations. Locations 37 (Rønne Banke) and 23 (Saaremaa) stand out as having the lowest shipping intensities in the radius of 20 km where on average only one vessel was present. Figure 2 also shows that in location 37 most of the ships occured in the distance interval 15–20 km. In contrast location 31 (Fehmarn Belt), was exposed to the most intensive shipping. In this location, on average two vessels were present at any given time within 5 km and more than eleven vessels within 20 km. Besides the shipping no other significant anthropogenic sound sources that could have affected the long-term sound levels were present during the monitoring in the selected locations.

## Results

### Analysis of spatial ambient noise variability

**Figure 3:**
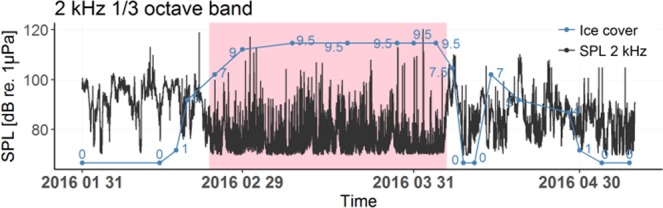
The effect of ice on the measured 2 kHz one-third octave band SPL in the Bothnian Bay BIAS sound monitoring location 11. The ice concentration is a fraction that expresses the sea surface ice cover in one-tenths (1/10). The complete coverage by ice corresponds to 10/10. The pink background highlights the time period when the ice concentration seems to affect the measured sound levels the most. During ice cover a wide selection of ice dynamics driven impulsive sounds is apparent. In this location the ship traffic intensity was relatively low but the recordings still contain the sounds of some ships breaking through ice.

Analysis of the spatial variability of ambient noise is based on the sound measurements in the selected BIAS sound monitoring locations. The underwater ambient sound was expected to be a mixture of the natural sounds, mostly caused by wind driven waves, and the anthropogenic sounds, mostly produced by commercial ship traffic. The spatial variability of the ambient sound arises from the spatial variability of the dominant sound sources as well as from the sound propagation conditions that are dependant on the water column properties, changing bathymetry and seabed substrates. As the Baltic is a shallow sea with average depth of 55 meters its temporal and spatial variability in the sound pressure level is expected to be larger than in the deep water. Also it is expected the high frequency sound contribution should be proportionally more important than in the deep water. The latter is explained by the cut-off phenomena reducing sound propagation in the lower frequencies^21,22^. In the Baltic Sea, the spatial variation is also caused by the occurrence of ice. While the Southern Baltic Sea is rarely frozen, the Bay of Bothnia and various other sea areas freeze annually. The extent of the ice cover can vary greatly with each year. The soundscape of the sea when covered with ice is expected to be very different when compared to the period when the sea is open^23^. This change was recorded in the middle of the Bay of Bothnia in the BIAS sound monitoring location 11 during the winter of 2016. Figure 3 shows 10 dB decrease in the SPL in the 2 kHz one-third octave band with the growing ice cover. The true decrease might be even higher as the recorders self-noise level restricted measuring the SPL lower than 70 dB re 1 *μ*Pa in this one-third octave band. The self-noise levels of the recorder are presented the methods section in Table 2. The presented ice cover data originated from the The Finnish meteorological Institute.

The tides are known to be a source of ‘pseudo-noise’ caused by turbulence around the hydrophone during tidal flow that contributes significantly at lower frequencies^16,24^. The Baltic Sea is shallow with brackish water and has been called a practically non-tidal sea by some authors^25^ and thus the pseudo-noise is not expected to occur. Another important part of the underwater soundscape is often contributed by marine animals. A well-known example in warm coastal waters is the impulsive biological noise produced by the snapping shrimp^26^. The biological contribution in the Baltic Sea long-term sound monitoring is expected to be mostly negligible, especially in its northern parts.

The annual underwater SPL values in different locations are concisely presentable by the estimated probability density function (PDF). A good way to compare different probability density functions of the one-third octave band SPL values is to compile them in the form of violin plots. As common for probability density functions, the area of each violin plot equals unity. The abscissa of the plots shows the probability for the occurrence of the SPL value displayed on the vertical axis. Various statistical measures are added to the violin plots for making them visually comparable and readable. The added statistical measures are the geometric mean (GM) and exceedance levels L5, L10, L25, L50, L75, L90, and L95. In the case of sound monitoring, exceedance level L95 is a low SPL value that is exceeded 95% of the time. Therefore, L95 can be related to the infrequent quieter natural sound levels. The exceedance level L5 is the SPL value that is exceeded only 5% of the time. In most cases it is related to occasional louder events when vessels pass close by the monitoring location. The statistical analysis presented hereafter was performed using bespoke software written in the programming language R^27^.

**Figure 4:**
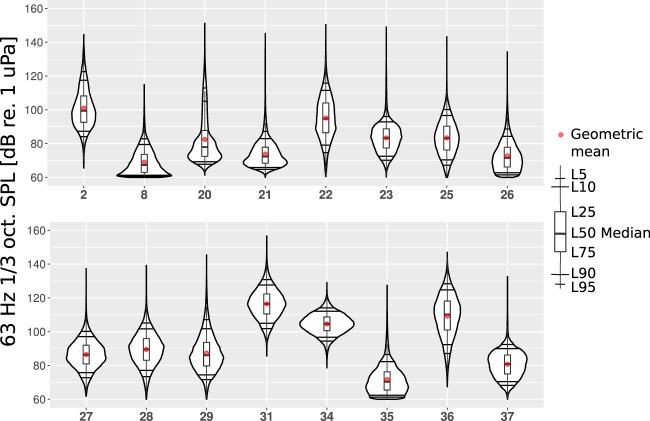
The estimated probability density functions of the measured SPL in sixteen different locations in the 63 Hz one-third octave band measured during the year 2014. The horizontal black lines represent L95, L90, L10, L5; the red dot marks the geometric mean (GM); the upper and the lower lines of the boxplot mark L25, L75; the thicker black line in the middle of the boxplot marks the median of the data.

**Figure 5:**
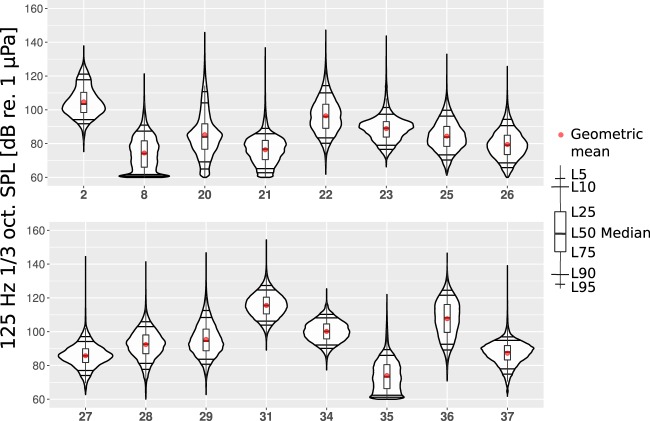
The annual estimated probability density functions of the SPL measured in sixteen different locations in the 125 Hz one-third octave band during the year 2014.

**Figure 6:**
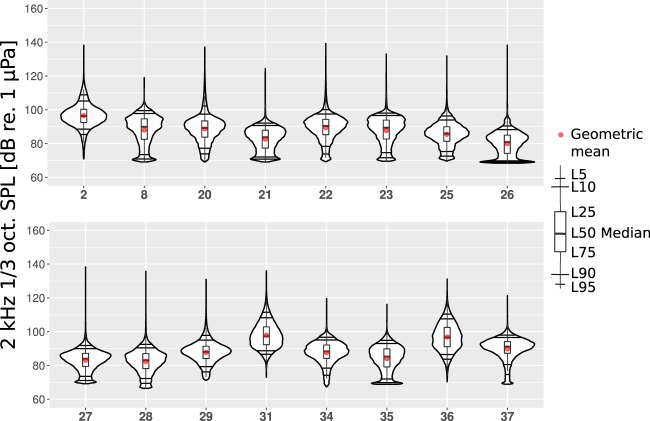
The annual estimated probability density functions of the SPL measured in sixteen different locations in the 2 kHz one-third octave band during the year 2014.

Figures 4–6 show the annual estimated PDFs as the violin plots for the selected monitoring locations and frequency bands. The SPL values in the two lower one-third octave bands lie mostly between 65 and 115 dB, while the 2 kHz one-third octave band SPL values are mostly between 70 and 100 dB re 1 *μ*Pa. In 63 Hz and 125 Hz one-third octave bands (Figs 4 and 5), the measured SPL values are highly dependent on the sound monitoring location. The difference between the median SPL values of the quietest location, no. 8 (Sea of Åland) and the loudest location, no. 31 (Fehmarn Belt), is around 50 dB for the 63 Hz one-third octave band and 40 dB for the 125 Hz one-third octave band. This observation confirms the prediction made by Urick^21^ about the expected large spatial variability of the low frequency sound levels in the shallow seas. Figure 6 shows that the spatial variability is much lower for the 2 kHz one-third octave band. The difference in the median SPL values within this one-third octave band when comparing locations 26 (Puck Bay) and 31 (Fehmarn Belt) is around 15 dB. At this higher frequency band, the natural sources, i.e., wind and surface waves, dominate over the shipping noise. The difference in the dominant sources is also indicated by the fact that the lowest median SPL value was in a different location for the 2 kHz one-third octave band when compared with the lower frequency bands. However, the monitoring locations where the shipping is intense, the 2 kHz one-third octave band levels are visibly higher (31, 34 and 36).

Most of the probability density functions do not follow a normal distribution. This is confirmed by the Shapiro-Wilk test of normality^28^. According to this test, the estimated PDFs of the 63 Hz one-third octave band SPL values (Fig. 4) from locations 27, 28, 31 are the closest to a normal distribution. For the 125 Hz one-third octave band SPL values (Fig. 5), only the PDF of location 31 is close to a normal distribution. In the absence of shipping, the non-normality of the PDFs can be anticipated as the wind speeds and significant wave heights usually follow a Weibull distribution^29,30^.

Figure 4 shows that half of the 63 Hz one-third octave band PDF-s (locations 2, 8, 20, 21, 22, 26, 29, 35) are asymmetric and visibly positively skewed as they have long thin upper tail and fatter lower tail. Only one PDF (location 36) has a visibly longer lower tail. Figure 5 shows that the 125 Hz one-third octave band PDFs are more symmetric when compared to the 63 Hz one-third octave band. In this octave band, the PDFs are still mostly positively skewed. The PDF of location 37 shows the largest negative skewness in the 125 Hz one-third octave band. The long and thin upper tails of the PDF plots are related to the rarely occurring loud events that in most cases are close passages of vessels. The differences in the upper ends of the tails indicate that the recorders were not subject to significant clipping. This was also confirmed by the clipping tests performed on the data during the processing. In contrast to the lower frequency bands, the 2 kHz one-third octave band PDFs in Fig. 6 show an average negative skew. This negative skew is largest for the PDF of monitoring location 37 where the ship traffic intensity is the lowest (Fig. 2). Figure 6 also shows that the largest positive skew is in location 31 that had the highest ship traffic intensity. The overall negative skew of the 2 kHz one-third octave band indicates the dominance of natural sound sources in this frequency range.

The lower tails of the violin plots are bounded by the limit imposed by the self-noise level of the SM2M recorder standard hydrophone with no gain (Table 2). In monitoring locations 8, 26 and 35, there might have occurred relatively low SPL values, often well below the self-noise level. These low levels could not have been recorded, instead, they were replaced by the recorders self-noise values. An example of a time series of the SPL values where the noise floor was reached is presented in Fig. 3. This “piling up” of self-noise values seems to cause the local maxima apparent in lower tails of the PDFs. The SPL values that are below the self-noise can be related to the lower sea states in sheltered waters. In order to avoid hitting the noise floor equipment with lower self-noise should be used. It is important to note that while the geometric mean values are affected by occurrence of self-noise, the median or exceedance levels L5, L10, L25 remain unaffected. When the effect of the self-noise level on the violin plots is acknowledged, they are still very useful for interpreting the soundscape at a specific location.

In spite of the apparent variability, some similarities in the PDFs can be found, mostly by regions and by similar conditions:

1. The highest annual SPL values in all the one-third octave bands were recorded near the Danish straits (locations 31, 36). This was somewhat expected, as Fig. 2 shows that these locations had also the highest shipping intensities among the monitoring locations. The depth at both of these monitoring locations was 27 meters. These monitoring locations were situated in the straits called Great Belt and Fehmarn Belt, which are frequently crossed by ferry-boats. Also, numerous cargo ships, tankers, including the largest ships, go through this deeper-watered route into the Baltic Proper. On top of that, seasonally, there is a high number of sailing and leisure boats.
2. The quietest locations with the lowest annual SPL values are more difficult to analyze when compared to the loudest. This is due to the aforementioned self-noise that limits the measurement of low sea state SPL values in the quietest locations. The sheltered location 26 is in the Bay of Puck (Poland) that is separated from the open sea by the Hel Peninsula. Location 35 was in a winding Danish strait the Little Belt. Although the annual 63 Hz and 2 kHz one-third octave band SPL values from these locations have relatively similar PDFs, they differ considerably in the 125 Hz one-third octave band. The quietest within the two lower frequency bands was location 8 (Sea of Åland). This does not follow any of the simple patterns as it is neither in sheltered waters nor with the lowest shipping intensity. The lowest levels in this location can partly be explained by the different data coverage, as the recordings are not available during the first four months of the year. Due to seasonal changes, these months are known to be the loudest ones. Still location 8 was the quietest when comparing the median SPL of the locations on monthly basis.
3. The locations in the Gulf of Finland (20 and 21) have similar annual PDFs for the SPL values. The waters are known to be mostly calmer than in the Baltic Proper, being protected from the South-Westward winds. Therefore, it is anticipated that the natural ambient SPL values are lower than in the Baltic Proper but higher than in sheltered waters. Locations 20 and 21 differ by the annual mean wind speed, which is lower in location 21. Also, they differ by their ship traffic intensity, which is higher in location 20, situated near busy crossing shipping lanes. Therefore, the annual SPL values at location 20 at all the frequency bands are expectedly higher when compared to location 21. The higher shipping intensity manifests itself as a longer and fatter upper tail and higher L5, L10 values in the PDFs for location 20.
4. The SPL levels in all the frequency bands for the monitoring locations in the Baltic Proper open sea conditions (2, 22, 23, 25, 27, 28, 29, 34, 37) have some considerable similarities and differences.

- In the Baltic Proper locations 37 and 23, overall ship traffic was lowest and the recorded sound may be considered mostly natural. The mean annual wind speed in these locations is almost the same, resulting in very similar PDFs.
- Locations 25, 27, 28 and 29 at the coast of Poland have quite similar PDFs. The differences in the PDFs follow loosely the differences in shipping intensities, wind speeds and depths. Locations 27 and 28 have both very low shipping intensities. The annual mean wind speed is higher and water is deeper in location 28. This anticipates the higher SPL values. When comparing location 29 with the previous locations, the shipping intensity is considerably higher, which means higher SPL levels in the 125 Hz and 2 kHz one-third octave bands. The very shallow depth of this location (12 meters) has probably constrained the higher SPL values due to the cut-off effect.
- Locations 2 and 22 had similar PDFs, both affected by higher annual mean wind speeds and higher intensities of shipping.

Similarities of the annual sound pressure level PDFs by regions have been presented previously in a study about the UK waters^16^. This study presented the 125 Hz one-third octave band PDFs of shorter deployments from ten monitoring locations in the North Sea.

**Figure 7:**
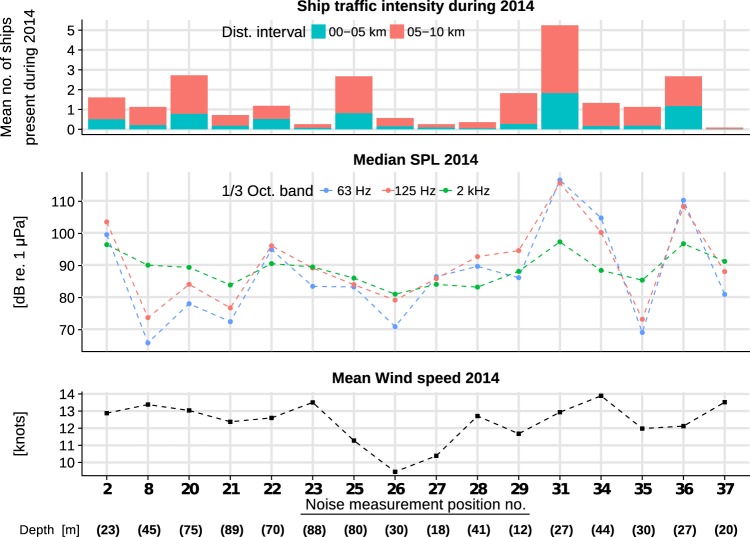
Median SPL one-third octave band levels at all monitoring locations in the middle along with the mean values of the ship traffic intensity on the top, mean wind speeds and the water depths in the bottom. The dashed lines joining the points are to be considered as aids for visualising the data and not as indicating the intermediate values between the locations. The wind speed data originates is the SMHI open-access data from MESAN weather analysis model.

For a better overview of the ensemble of the monitoring locations, Fig. 7 presents the annual median results for all one-third octave bands together with the depths, annually averaged shipping intensity and wind speeds. Comparison of the graphs shows the following:

- The annual median SPL values of 63 Hz and 125 Hz one-third octave bands have strong correlation. The 125 Hz one-third octave band annual median SPL values in monitoring locations are on average 3 dB higher.
- The 2 kHz one-third octave band annual median SPL values correlate weakly with the lower frequency octave bands.
- Some patterns can be noted, when looking at which of the annual median one-third octave band SPL value was highest in a monitoring location. For half of the locations (8, 20, 21, 23, 25, 26, 35, 37), the highest annual median SPL value is in the 2 kHz one-third octave band, followed by the 125 Hz and 63 Hz one-third octave bands. This order was reversed in three locations (31, 34, 36). The reversal hints at some significant differences in the overall recorded sounds spectra in locations where ship traffic is very intense.
- As expected, the two lower frequency band median SPL values seem to be more affected by the ship traffic intensity than is the 2 kHz one-third octave band.
- The median 2 kHz one-third octave band values in locations with very intense traffic are higher. Otherwise, some dependence on mean wind speeds can be noted.

## Analysis of Temporal Variability in Ambient Noise

The temporal variability in the ambient noise of the Baltic Sea is known to be periodic at different time scales. Diel variations have been measured that were related to vertical migration of marine organisms in the southern part of the Baltic Sea^31^. Simultaneously AIS data analysis has shown that some shipping activities have also diel patterns. This is understandable, as ferry boats have daily schedules, fishing boats have their daily routines and so do the leisure boats and sailing ships. Preliminary analysis of our data has shown significant diel variations in the SPL values. In locations with more intense ship traffic these variations coincided with the diel changes in the number of ships present around a monitoring location.

The seasonal variability of the recorded sound is mostly related to the periodic variations in the sound speed profile of the water column. The upper layers of the water column get warmer during summer months. As a result, the sound waves refract downwards and reach the bottom, causing faster loss of the propagating acoustic energy^32^. The wind speeds and wave heights in the Baltic Sea are also known to have a seasonal periodicity, both being higher during the winter months^33^. Besides the natural sources of seasonality, some ship types have also seasonal occurrence patterns.

The seasonal variability in SPL values in the southern Baltic Sea has been measured to be in the range of 12 dB^34^, or 10–15 dB^31^ with greater levels during the winter. For the seasonal variability, the monthly median SPL values in all the selected monitoring locations and frequency bands were calculated and compared. As was expected, the highest monthly medians were recorded during the winter and the lowest in the summer months. The minimum in the monthly medians for all the one-third octave bands in most positions was recorded in July. The month with the highest median varied according to the frequency band. In the 63 Hz and 2 kHz one-third octave bands, the loudest month in most locations was December. As for the 125 Hz one-third octave band, in most locations, the loudest months were January and February. The difference between the medians of the loudest and the quietest was on average 10 dB. In the monitoring locations with intense shipping, the monthly medians changed less throughout the year when compared to other locations. For example, location 31 with most intense shipping had only 2 dB seasonal difference in the 63 Hz one-third octave band, 3 dB in the 125 Hz one-third octave band and 6 dB in the 2 kHz one-third octave band.

Comparison of two years of sound monitoring results in a location has the potential to estimate the plausibility of finding long-term trends. In some of the selected monitoring locations, the deployments continued after the end of the BIAS project. Two of these were locations 20 and 26. Location 20 is in the Gulf of Finland near the intersection of busy eastward shipping lane to St Petersbourg and northward ferry route between Tallinn and Helsinki. After 2014, the next yearly monitoring in this location was performed from November 2015 until October 2016. Location 26 is in the sheltered Polish waters of the Bay of Puck over 10 km to the north from the port of Gdynia.

**Figure 8:**
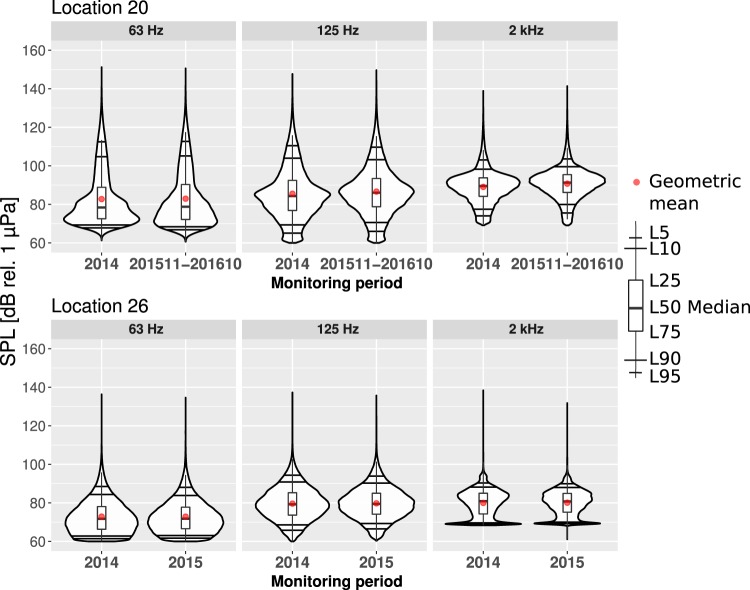
Estimated probability density functions of the measured SPL at three different one-third octave bands for the measurement location 20 (Tallinn, EST) and location 26 (Puck Bay, POL) for two monitoring periods.

Figure 8 presents the comparison of two years of sound monitoring results in locations 20 and 26. As can be seen, the results of two consecutive annual periods are similar for all three frequency bands. This hints that the yearly averaged soundscape in a given location remains relatively unchanged. Even if the seasonal variability of noise is high, the overall annual values are similarly distributed from one year to another. This implies that sound monitoring has to cover most of the year to give a representative estimate to the prevailing SPL values in a location. The yearly SPL values serve as a baseline for this location, against which to compare any measurements in the same location in the following years. The results of comparing the two years with the Mann-Whitney U test (significant if p-value < 0.05) are as follows:

- In location 20, the 63 Hz one-third octave band had no statistically significant change in the annual SPL values, while in the 125 Hz and 2 kHz one-third octave bands there was a statistically significant increase in the SPL values. The annual median value in the 125 Hz one-third and 2 kHz octave bands was about 1.7 dB higher in the second monitoring period.
- In location 26 for all the one-third octave bands, there was no significant change in the annual SPL values. The difference in the medians between the two monitoring periods was less than 0.25 dB.

Similar low interannual variability has been previously observed at some monitoring locations in the North Sea^16^.

**Figure 9:**
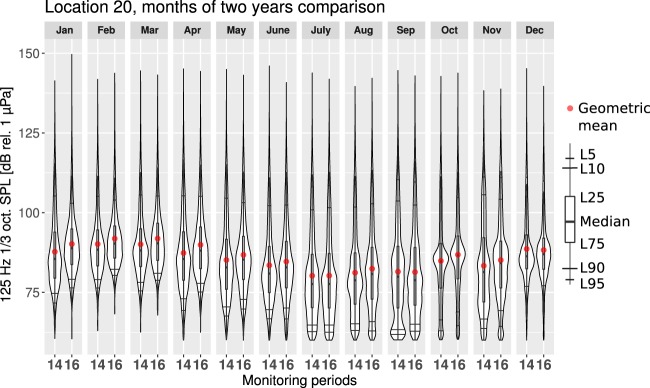
Comparison between monthly estimated PDFs from two separate monitoring periods in the 125 Hz one-third octave band for the measurement location 20 (Tallinn). The number 14 on the x-axis marks the monitoring period from from 1 January 2014 to 31 December 2014 and the number 16 marks monitoring period from from 1 November 2015 to 31 October 2016.

The differences in the seasonal changes of the SPL values between the two years can be better exemplified by comparing the monthly estimated PDFs. Figure 9 shows that the monthly SPL values from two different monitoring periods recorded in one location have similar distributions. For most of the months, the second monitoring period has higher median SPL values. Although the loudest month remained unchanged, being still February, the median value was higher in the second monitoring period. Figure 9 also shows that during the winter months, the lower SPL values were above the self-noise level of the marine recorder. From June to November, the SPL values seem to have been occasionally below the self-noise level. Thus, due to seasonality, the previously discussed effect of self-noise “piling up” in the lower tails of the PDFs is more likely present in the summer months. Interannual comparison on the monthly basis reaffirms that the variability is growing with the reduction of the averaging time period.

## Conclusions

Baltic Sea underwater ambient sound pressure levels were measured in monitoring programmes running since 2014. These monitoring programmes have been pioneering in terms of their coordinated cross-bordering effort, longevity and spatial coverage. The collected data offer an opportunity to analyse the spatial and temporal variability of the sound. Analysis was restricted to 16 selected monitoring locations out of the total 36 in the BIAS-project. The choice was made due to the annual data coverage and the aim to represent different natural conditions and ship traffic intensities in the Baltic Sea. The presented analysis also revealed that any future monitoring of noise in calm sheltered waters should consider the use of recording equipment with lower self-noise.

The annual SPL values from the different monitoring locations show high variability with some clear regional similarities. The difference between the loudest and quietest location was almost 50 dB in 63 Hz one-third octave band and 40 dB in 125 Hz one-third octave band. The locations with the lowest recorded annual median SPL were recorded in calm sheltered waters, while the highest annual median SPL values were in the Danish straits where a lot of vessels are present at any given time. The annual estimated probability density functions (PDFs) of the two lower frequency bands were often positively skewed, especially for the locations with low median SPL values. For most of the locations, the recorded SPL had clear relation with prevailing wave/wind weather and shipping intensity. However, in some locations, the relation was not so clear and the interplay of the sound propagation conditions along with sound sources should be further investigated.

When monitoring noise at the same locations for two yearly periods, the overall sound levels of the two periods were found to be very similar. This was confirmed in two separate locations, one in the Gulf of Finland and another in the Bay of Puck. The temporal variability was shown to be highly dependent on the time scale. This was illustrated by the different monthly SPL values for two consecutive annual periods contrasting with the similar annual values. This shows the decrease of variability with the increase of averaging time period. This low variability of the annual PDFs indicates that it is a good measure of the baseline SPL values at a given location. The seasonal variations of sound pressure levels were found to be in the range of 10 dB with lower variation in locations with a heavier traffic. This indicates that in many areas of the Baltic Sea shipping already contributes significantly to the prevailing ambient sound levels. However, more effort should be made for better assessing the contributions made by ships by differentiating between monitored sound levels in presence and absence of ships.

## Methods

### Sound pressure monitoring technique

**Figure 10:**
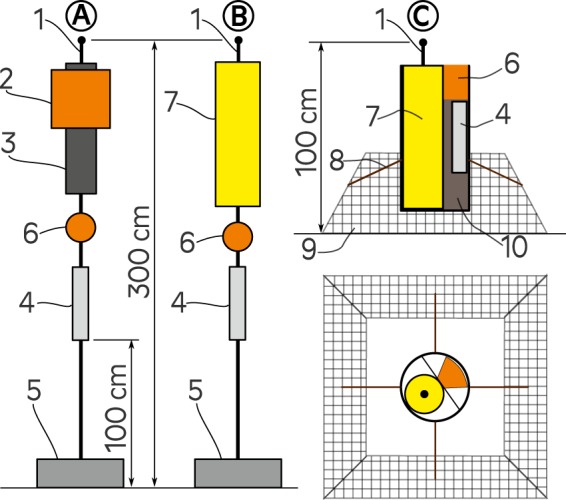
Sketch of the three BIAS standard rigs marked with letters A,B,C; 1-hydrophone, 2-extra buoyancy, 3-DSG Ocean recorder, 4-acoustic releaser, 5-ballast (min 20 kg wet weight), 6-buoy, 7-SM2M recorder, 8-rope, 9-steel grid cage, 10-rope container^35^.

The presented measurements were performed following the measurement and signal processing standards developed for the BIAS-project^35,36^. In order to assure the comparability between different countries the use of standards in measurements was essential. In the BIAS-project, two different autonomous recording systems were used for monitoring the continuous underwater sound: the DSG Ocean marine recorder manufactured by Loggerhead Instruments and the SM2M manufactured by Wildlife Acoustics^37^. Figure 10 presents the setups of the standard measurement rigs. The DSG Ocean marine recorder was used in the rig design marked with letter **A**. This design was deployed for some periods in sound monitoring locations marked with the triangular markers in Fig. 1. Letter **B** in Fig. 10 marks the most used rig design with the SM2M recorder. The exceptions were the rigs deployed in the Polish waters which had an alternative design marked with **C**. The probability of losing the rig due to trawling was high there and additional protection was needed. In the alternative rig-design, the hydrophone is located 1 m above the seafloor instead of 3 m. The recording system is surrounded by a protective structure made up of pyramid-shaped steel frame. This configuration allows the sensor pod to tilt and the trawl net to slip over the rig. A plastic tube serves as a housing that protects the recorder, acoustic releaser and rope container against direct impact from trawls^35^. The presented rig designs proved suitable for monitoring in the Baltic Sea. Although it must be noted that they may not be optimal for locations with high tidal flow.

**Table 2:** Evaluated self-noise and spectral densities of the SM2M recorder in the one-third octave bands 63 Hz, 125 Hz and 2 kHz.

One-third Octave central freq.	63 Hz	125 Hz	2 kHz
Self-noise [dB re 1 *μ*Pa]	63	65	70
Spectral density [dB re 1 *μ*Pa^2^/Hz]	51	50	43

In all the monitoring locations chosen for this study, the ambient sound was recorded during the whole year of 2014 and therefore, the annual data are comparable. Duty cycles in the different monitoring locations varied from 20 to 59 minutes per hour. Data exclusion tests with the 59 minute duty cycle recordings did not indicate to any significant effect on the long term monitoring results. The standard SM2M marine recorder allows measurements with sampling frequencies from 4 kHz to 96 kHz and a bit depth of 16-bits. Most of the measurements were made with a sampling frequency *f*_*s*_ = 32 kHz. In addition to the indicator one-third octave bands, centered at 63 Hz and 125 Hz, a one-third octave band centered at 2 kHz and a broadband 10 Hz–10 kHz were monitored in the BIAS-project. The 2 kHz one-third octave band was added as it is more relevant for marine mammals, which have poor hearing abilities at lower frequencies.

The frequency response of the SM2M can be considered to be relatively flat, being +/−2 dB of the rated sensitivity in the frequency range that spans all the one-third octave bands of interest. This enables the sensitivity *M*_*f*_ of the instrument to be handled as a single number. All acoustic recorders were point calibrated (frequency 250 Hz) with a pistophone before the first deployment and after the last retrieval. Within the range of +/−1 dB, no significant change in sensitivities was observed. Table 2 lists the evaluated self-noise of the SM2M recorder in the one-third octave bands.

### Recorded sound pressure data processing

The Good Practice Guide for Underwater Noise Measurement^10^ states that: “The metric most suitable for quantifying the continuous sounds, is the Sound Pressure Level (SPL)”. The SPL is defined among other entities in the ISO standard^38^ as

$${L}_{p}=10{\log }_{10}\frac{{\hat{p}}^{2}}{{p}_{0}^{2}},$$

where *p*_0_ is the reference value of sound pressure which in water is *p*_0_ = 1 *μ*Pa. The ${\hat{p}}^{2}$ is the mean-square sound pressure that is defined as

$$\hat{p}=\frac{1}{{t}_{2}-{t}_{1}}{\int }_{{t}_{1}}^{{t}_{2}}\,{p}^{2}(t)\,dt$$

where *p*(*t*) is the sound pressure, and *t*_1_ and *t*_2_ are the start and end times, respectively. As already addressed in the introduction, the indicator D11 criterion for continuous low-frequency sound^11^ specifies that the average sound level (re 1 *μ*Pa root-mean-square RMS) should be presented in the one-third octave bands with center frequencies of 63 Hz and 125 Hz. In the BIAS-project, the average of the sound level in the one-third octave band was determined as the geometric mean of 20 consecutive one-second averaged one-third octave band root-mean-square sound pressure levels, i.e. computationally approximately equivalent to RMS-average over the 20 seconds. The reason behind this granularity in the data resolution was the consideration of making the data publicly shareable. Some national authorities considered a finer resolution to compromise their national security interests.

The SM2M and DSG recorders, as various other marine submersible recorders, digitise the measured sound pressure and store it as discrete values in *.wav*-file format. As an initial step, the discrete 16-bit values are scaled to volts. The root-mean-square of discrete values is calculated with the following formula:

$$\hat{x}=\sqrt{\frac{1}{N}\mathop{\sum }\limits_{n=1}^{N}\,{x}^{2}[n]}.$$

This can be rewritten by applying the Parseval’s theorem. For a sampled value *x*[*n*] = *x*[*t* = *NT*], where *T* is the sampling period (equal to the reciprocal of the sampling frequency)

$$\mathop{\sum }\limits_{n=1}^{N}\,{x}^{2}[n]=\frac{1}{N}\mathop{\sum }\limits_{m=1}^{N}\,{|X[m]|}^{2},$$

where *X*[*m*] is the Discrete Fourier Transform (DFT) of *x*[*n*], *N* is the number of samples and DFT coefficients. In practice, the root-mean-square of the discrete samples can be obtained from the sum of the frequency amplitude spectrum. Therefore, the root-mean-square of a discrete valued variable can be calculated from

$$\hat{x}=\sqrt{\frac{1}{{N}^{2}}\mathop{\sum }\limits_{m=1}^{N}\,{|X[m]|}^{2}}$$

No window functions were applied to the data prior to the estimation of the DFT since it did not improve the statistical estimates. If the sensitivity |*M*_*h*_| of the measurement system is known (the gain was *G* = 1 and the discrete voltage values *v*[*n*] were also known from scaling) the SPL was calculated from the recorded bits as

$${L}_{p}=|{M}_{h}|+10{\log }_{10}{\hat{v}}^{2}=|{M}_{h}|+10{\log }_{10}(\frac{1}{{N}^{2}}\mathop{\sum }\limits_{m=1}^{N}\,{|V[m]|}^{2}),$$

where $\hat{v}$ is the RMS voltage of *N* samples and *V*[*m*] the FFT of *v*[*n*]. If the one-third octave band SPL values are calculated, the last expression becomes

$${L}_{{p}_{1/3}}=|{M}_{h}|+10{\log }_{10}(\frac{1}{{N}^{2}}\mathop{\sum }\limits_{m={k}_{1}}^{{k}_{2}}\,{|V[m]|}^{2}),$$

where *k*_1_ and *k*_2_ are the indices corresponding to a given one-third octave bands lower and upper frequencies.

The BIAS-project established Quality Assurance (QA) in order to assure equal data quality among the BIAS partners. An inter-comparative analysis, “ring-test”, was carried out among all the participating countries. Identical sound samples were processed by all beneficiaries and the results compared. The sample data were processed by the named experts from each of the participating countries. The test checked the sample length, portion of clipping and SPL values in all the monitored frequency bands. The ring tests were found to be a useful tool for the QA. All discrepancies were investigated and at the end of the testing, the results of all the six countries were in agreement with one another.

## Data Availability

The one-third octave band SPL values, AIS data and other datasets analysed during the current study are available from the corresponding author on reasonable request. The recorded raw data are not publicly available due to confidentiality reasons.
